# Internal and external information in error processing

**DOI:** 10.1186/1471-2202-9-33

**Published:** 2008-03-25

**Authors:** Marcus Heldmann, Jascha Rüsseler, Thomas F Münte

**Affiliations:** 1 Department of Neurology II, Otto-von-Guericke University Magdeburg, 39120 Magdeburg, Germany; 2 Department of Psychology II, Otto-von-Guericke University Magdeburg, 39106 Magdeburg, Germany

## Abstract

**Background:**

The use of self-generated and externally provided information in performance monitoring is reflected by the appearance of error-related and feedback-related negativities (ERN and FRN), respectively. Several authors proposed that ERN and FRN are supported by similar neural mechanisms residing in the anterior cingulate cortex (ACC) and the mesolimbic dopaminergic system. The present study is aimed to test the functional relationship between ERN and FRN. Using an Eriksen-Flanker task with a moving response deadline we tested 17 young healthy subjects. Subjects received feedback with respect to their response accuracy and response speed. To fulfill both requirements of the task, they had to press the correct button and had to respond in time to give a valid response.

**Results:**

When performance monitoring based on self-generated information was sufficient to detect a criterion violation an ERN was released, while the subsequent feedback became redundant and therefore failed to trigger an FRN. In contrast, an FRN was released if the feedback contained information which was not available before and action monitoring processes based on self-generated information failed to detect an error.

**Conclusion:**

The described pattern of results indicates a functional interrelationship of response and feedback related negativities in performance monitoring.

## Background

To adapt ongoing behavior in a changing world human beings have to compare performed actions against their intended outcome. This comparison might be based on internally as well as on externally represented information. Using event related brain potentials (ERP, see [1]) time locked to motor responses, performance monitoring processes based on internal information are reflected in a sharp negative deflection with a peak latency of 70–100 ms after erroneous responses and a fronto-central maximum. Several functional interpretations have been advanced for this error-(related) negativity (ERN or Ne [2,3]) of which the following are pertinent here: The conflict-monitoring approach holds that the amplitude of the ERN varies as a function of the degree of conflict between two or more prepotent response tendencies. In contrast the error-monitoring theory postulates that the ERN is related to the detection of the erroneous response. If the ERN indeed signals the detection of an error, the question arises in which way its amplitude is related to the consciousness of having made an error. Two different, but partially overlapping concepts addressing this topic: error awareness and error certainty. By using antisaccade [4,5] or GoNogo inhibition tasks [6] it was shown that the ERN could occur without conscious error awareness. Imaging studies supporting this view showing no activation differences between aware and unaware errors in the corresponding error processing anatomical structures (anterior cingulated cortex [7], rostral cingulate zone [8]). In contrast to error awareness there is some evidence that the subjective certainty of having made an error influences the ERN amplitude. Scheffers and Coles [9] reported varying ERN amplitudes according to the participants' rating of being correct or not. Errors for which subjects were uncertain about their incorrect response resulted in a decreased ERN amplitude compared to "sure" incorrect responses. Pailing and Segalowitz [10] reported a similar pattern of results by inducing uncertainty about the subjects' task performance. In their Flanker task a decreased ERN was observed when subjects had to perform a secondary task at the same time. The linear relationship of error certainty and ERN amplitude was shown by results of Luu et al. [11] who defined two criteria for making a correct response, (i) pressing the correct button and (ii) responding faster than an individually adjusted response deadline. While the ERN to incorrect button presses was largest, the amplitude after correct but too slow reactions depended on response speed: the slower the response, the larger the ERN-amplitude and, presumably, the stronger the subject's certainty of a time-out error.

The use of externally provided information on performance is reflected in the feedback related negativity (FRN, or feedback ERN). Miltner and colleagues [12] were the first to describe a negativity peaking around 250 ms after feedback presentation with a maximum over the midfrontal scalp (see also [13]). The functional meaning of the FRN is assumed to be very similar to the ERN: non-satisfying outcomes of relevant events are leading to a negative deflection compared to positive outcomes. Beyond this there is some disagreement concerning FRN amplitude variations: While some authors reported FRN amplitude variations according to different amounts of reward or punishment [14-18] others showed that the FRN's amplitude reflects reward and punishment in a more binary fashion [19-24]: non-satisfying, punishing or non-rewarding events resulted in a pronounced negative amplitude irrespective of the indicated amount of punishment or non-reward. In line with this view, neutral feedback has to be considered as a non-satisfying event leading to an FRN, which can only be differentiated from positive, but not from negative feedback ([21], but see [25] for a different interpretation).

Most studies investigating the functional meaning of the FRN did this without taking its relationship to the ERN into account (for a review see [26]). By contrast, the groups around Holroyd, Coles and Nieuwenhuis conducted a series of studies using probabilistic learning tasks to determine the relationship between ERN and FRN [13,27,28]. This paradigm required to learn the correct stimulus response mapping via trial-to-trial feedback. Holroyd and Coles [27] and Nieuwenhuis et al. [13,28] used different levels of feedback validity to manipulate the predictive value of a given response. According to these authors the predictive value refers to the subjects' ability to infer the outcome of an action – in the studies cited above the resulting feedback – on the basis of the given response. If the predictive value of a response is high, internally available information enables the organisms' action monitoring processes to indicate an error. These processes fail to detect an error in situations with low predictive values. Therefore externally provided information has to be used for adapting ongoing behavior. As predicted, Holroyd and Coles [27] and Nieuwenhuis et al. [13] reported increased ERN, but nearly absent FRN amplitudes in conditions with high predictive values. In conditions with non-informative feedback the predictive value was low, therefore decreased ERN, but increased FRN amplitudes were shown. This inverse relationship in the appearance of ERN and FRN indicated that a non-rewarding event per se is not sufficient to release an FRN, the feedback has also to contain relevant information not available before. More generally within the stimulus – response-feedback sequence only the first error indicating event results in a negative deflection within the ERPs.

In the present study the predictive value of a given response was varied by the apparentness of having made an error. Similar to the paradigm used by Luu and colleagues [11] we applied an Eriksen-Flanker task [29] with an additional response deadline procedure. Participants had to fulfill two criteria for making a correct response: (1) pressing the correct button and (2) performing this response with adequate speed. While the first criterion, correctness of the current button press, had a high predictive value, the predictive value of the second criterion, performing the button press with adequate speed, varied according to the subjects' ability to detect a passing of the valid response deadline. Since responses with correct button presses, but very slow reaction times could be detected by the subjects they had a high predictive value. In contrast the predictive value of reactions with response times passing the valid deadline slightly was low. While Luu and colleagues [11] defined the deadline based on the subjects' performance during the training session only, we adjusted the individual response deadline after each block. Thus, our subjects were informed more precisely about their actual performance by receiving three kinds of symbolic feedback: (1) correct button press and response speed faster than the valid deadline, (2) correct button press, but response speed slower than the valid deadline and (3) incorrect button press with no information regarding the response speed.

Based on previous findings [13,27] we predicted for responses with correct button selections but an obvious violation of the response deadline increased response-locked negativities, but no variations for the feedback-locked component. When subjects made a correct button press missing the response deadline narrowly the given response had no predictive value. In this case we expected an inverted ERN-FRN relationship: no variation for the response-locked component, but an increase in the feedback-related negativity.

## Methods

### Participants

Twenty-one healthy right-handed students (11 women, mean age 23.1) participated in the experiment after giving informed consent. All persons had normal or corrected to normal vision, were neurologically healthy and were compensated either with course credits or with 32.50 Euro. Due to equipment malfunction or massive ocular artifacts four subjects were excluded leaving seventeen subjects for the reported analyses. The study protocol was approved by the ethics committee of Magdeburg University.

### General procedure

Participants were seated in a comfortable chair in front of a 19"-CRT monitor. A modified computer mouse was positioned under each index finger as a response device. The experiment consisted of two identical sessions which took place within one week. Every session started with 100 practice trials without feedback followed by the demonstration and explanation of the feedback stimuli. Thereafter the experiment started comprising twelve blocks of 100 trials each. Between the blocks breaks of 30 s were taken.

### Task

**Figure 1:**
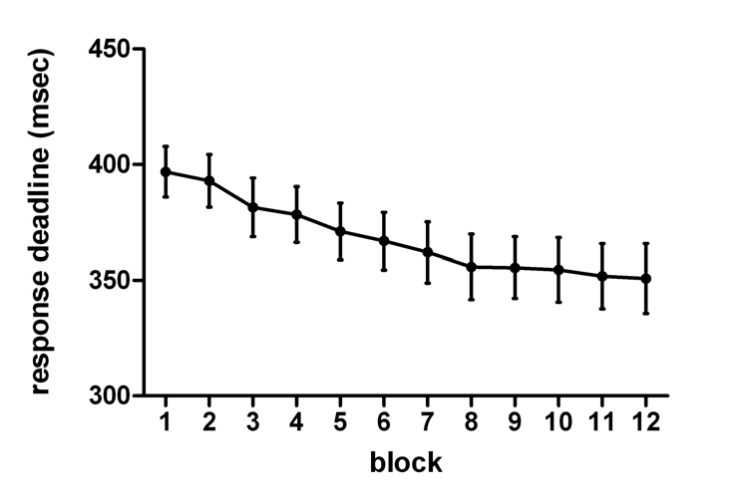
**Response deadlines per block**. Mean response deadlines per block.

An Eriksen Flanker task [29] was used with stimuli comprising one of the following letter strings: HHHHH, SSSSS, SSHSS or HHSHH. Participants were required to focus on the center letter and to signal whether this letter was an „H“ or an „S“ by pressing a mouse button with the right or left index finger respectively. Strings with identical letters ("congruent") were presented in 40%, strings with different letters (e.g. HHSHH, "incongruent") in 60% of all trials. Each flanker stimulus was followed by a feedback stimulus consisting of a colored square (orange, blue or pink), indicating whether the preceding reaction was "correct and in time", "correct but out of time" or "false". Keys and colors were counterbalanced across subjects. The in time/out of time feedback criterion depended on a moving response deadline. This deadline was defined by the upper border of the 6th decile of the reaction time (RT) distribution of the previous block. Caused by this adaptive procedure the response deadlines became more demanding over time (see figure 1). Furthermore, response times slower than 1000 ms were defined as "no reaction" and hence received no feedback. When being in time, they received the feedback of being too slow. If their responses were out of time, the feedback stimulus „in time“ was shown. Additionally, in 5% of all feedback stimuli subjects received an incorrect feedback with respect to the time criterion. This was done to test the influence of incorrect feedback on the proposed relationship of response and feedback related negativities. Unfortunately too many EEG artifacts disallow a reliable analysis of this condition. The flanker stimuli were presented for 100 ms, the feedback stimuli for 200 ms. The SOA flanker-feedback stimulus was randomized between 1000 and 1500 ms, the SOA feedback-flanker stimulus between 1400 and 1900 ms. All stimuli were presented slightly above a constantly visible line (width 1.1° visual angle) in the middle of the monitor. Flanker stimuli subtended 3.4°/1.1° in width/heigth, the feedback stimuli subtended 2.9° by 2.9°.

### EEG-recording and data analysis

The electroencephalogram was recorded from 29 tin electrodes mounted in an elastic cap and placed according to the international 10–20 system. EEG was rereferenced offline to the mean activity of the left and right mastoid. To enable the offline rejection of eye movement artifacts, horizontal and vertical electrooculograms (EOG) were recorded using bipolar montages. All channels were amplified (bandpass 0.05 – 30 Hz) and digitized with 4 ms resolution. Using individualized amplitude criteria on the eye channels, trials with eye movement artifacts were excluded from the analysis.

Trials with RTs faster than the response deadline were classified as „early“ (EA), trials with RTs slower than the response deadline as "early late" (EL) or "late late" (LL) trials. For each block this differentiation was made by computing the median of all RTs exceeding the individual response deadline. Thus, EL trials were trials slower than the deadline but faster than the median, while LL trials were those trials exceeding the median RT of the late responses. Additionally, for each time bin trials were categorized into correct and incorrect responses. Only the incorrect trials of the EA bin were used for analysis of the effects of choice errors, while in the remainder of the paper EA, EL and LL conditions will refer to responses with correct button selection only. After categorizing trials according to the above criteria response-locked (epoch length 900 ms, 300 ms baseline) and feedback-locked (epoch length 700 ms, 100 ms baseline) averages were calculated for each subject. To remove slow wave potentials like the P3 [30] data were subjected to a 4 – 12 Hz band pass filter (half amplitude cut-off, the effect of a 4–12 Hz filter can be seen in figure 3). Finally, filtered averages of all seventeen subjects were collapsed to calculate the grand averages. For all ANOVAs mean amplitudes (0–120 ms for response locked, 260–320 ms for feedback locked data) were used. In order to test the influence of incorrect button press responses on ERN and FRN-amplitude by itself an ANOVA with the factors correct/incorrect (2 levels) and electrode site (Fz, Cz, Pz) was performed for response- and feedback locked data of the early (EA) RT-bin. To assess the influence of the subjects' ability to detect reaction time errors of correct button press responses on ERN- and FRN-amplitudes, ANOVAs with the factors RT-bin (EA, EL, LL) and electrode site (Fz, Cz, Pz) were performed for the two components. To confirm the assumed interaction between ERN/FRN amplitude and availability of performance related information we calculated a repeated measures ANOVA with the factors electrode site (levels Fz, Cz), ERN/FRN (2 levels) and the factor RT-bin (3 levels). Prior to this analysis data were subjected to a vector-normalization procedure [31,32] to remove overall amplitude differences of FRN and ERN. The Huynh-Feldt epsilon coefficient was applied to correct ANOVAs for non-sphericity. The original degrees of freedom but corrected p-values will be reported.

## Results

### Behavioral data

The mean reaction times for correct responses were 333 ms (EA, SD 45 ms), 399 ms (EL, SD 52 ms) and 496 ms (LL, SD 59 ms). For the incorrect responses of the EA time bin mean reaction time was 311 ms (SD 48 ms). In 58% of all correct responses subjects reacted faster than the valid deadline, while 21% of all reaction times were classified as EL and as LL responses each.

### EEG-data

**Figure 2:**
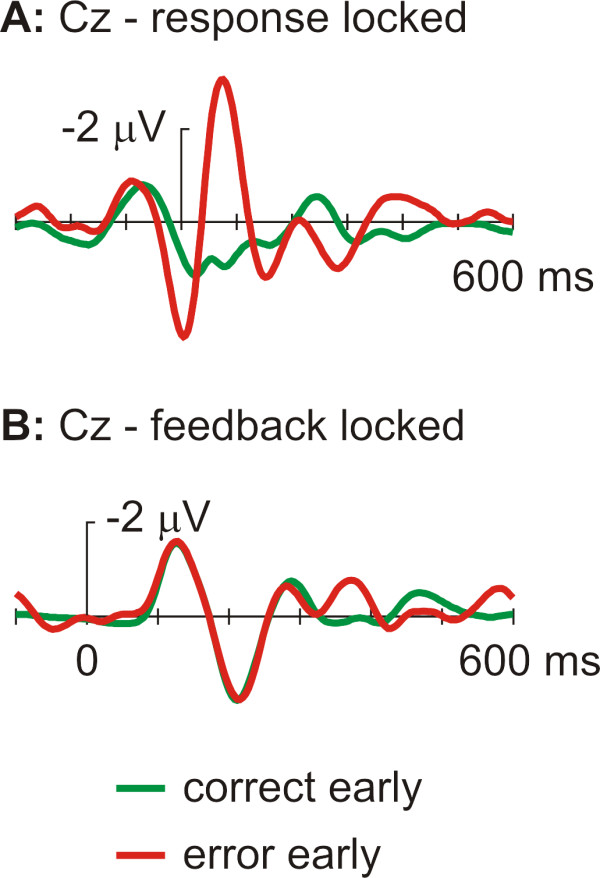
**Response and feedback locked ERPs for in-time responses**. Grand averages (Bandpass filtered 4–12 Hz) at electrode site Cz for responses that were faster than the deadline: A) time locked to correct and erroneous responses, B) time locked to feedback stimuli.

To test the influence of erroneous button press responses we looked at the ERPs of the RT-bin EA first (see figure 2). The analysis of the response locked ERPs (repeated measures ANOVA with the factors correct/incorrect F(1,16) = 39.9; electrode site F(2,32) = 33.9; interaction F(2,32) = 12.9; all p < 0.001) revealed a clear ERN (see figure 2A), which was at Cz significantly different (t(16) = 6.0, p < 0.001) from correct responses within this time bin. In contrast, using a repeated measures ANOVA (factors correct/incorrect (2 levels) and electrode site (levels Fz, Cz, Pz)), corresponding ERPs time locked to the feedback stimuli (FRN, see figure 2B) showed neither a significant correct/incorrect main effect (F(1,16) = 1.0, n.s) nor a significant correct/incorrect × electrode site interaction (F(2,32) = 3.14, n.s.). Only the electrode site main effect was significant (F(2,32) = 4.52, p = 0.045).

**Figure 3:**
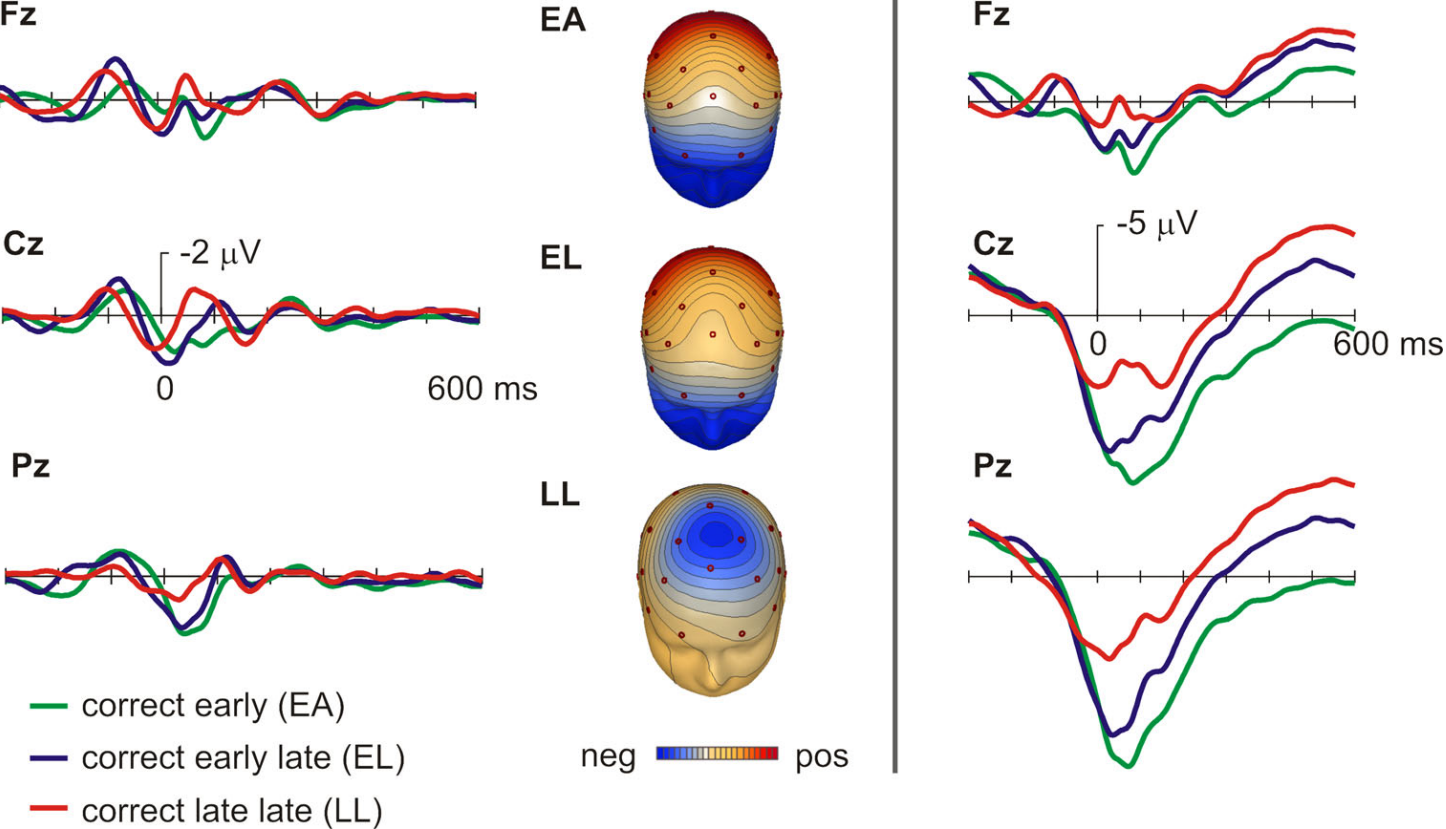
**ERPs time locked to correct responses for all timing conditions**. Left panel: Bandpass filtered 4–12 Hz grand averages and isovoltage maps for correct responses of all time conditions (see text for details). Color scale represents steps of 0.1 microV. Right panel: Grand averages for identical conditions without bandpass filtering.

**Figure 4:**
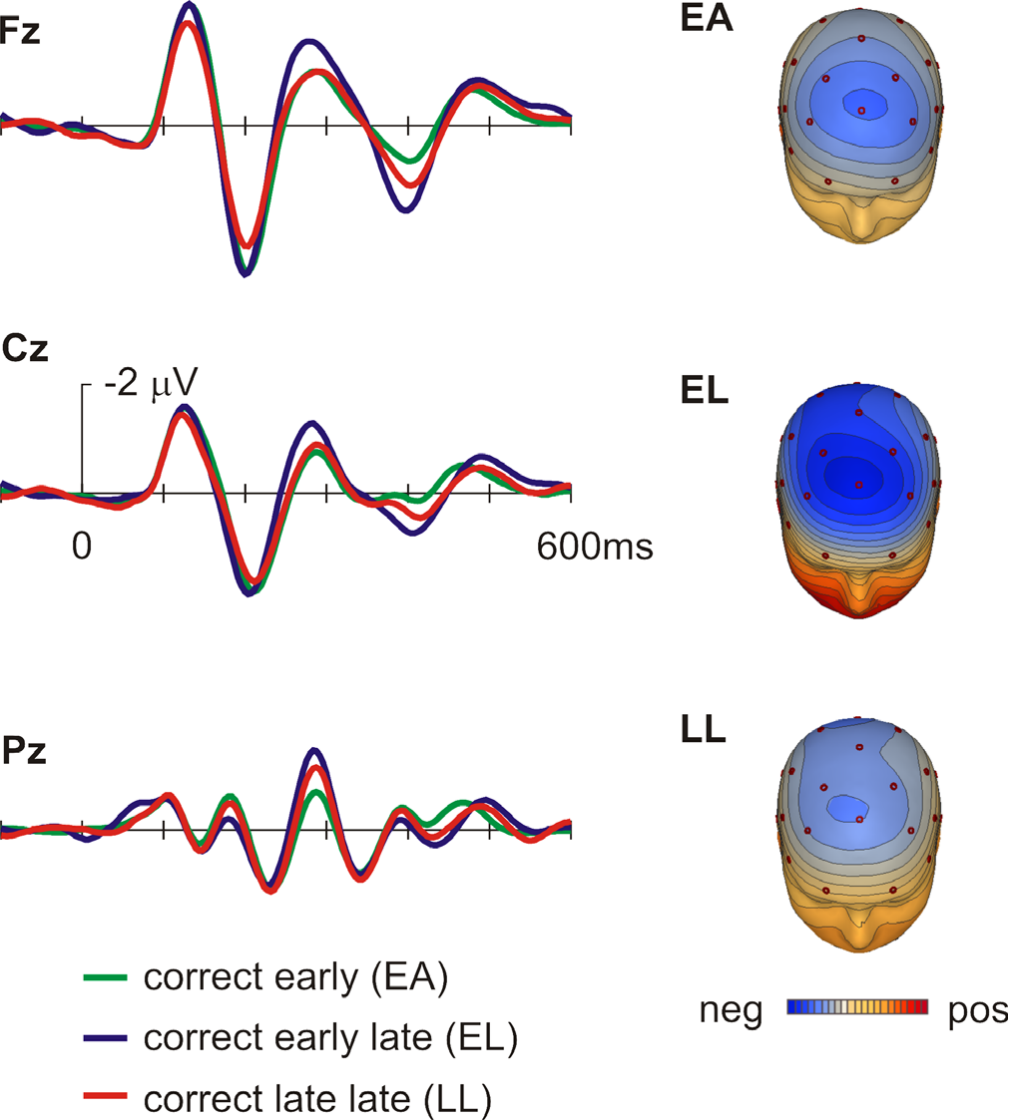
**ERPs time locked to feedback stimuli**. Grand averages (bandpass filtered 4–12 Hz) and isovoltage maps for ERPs time-locked to the feedback stimuli (see text for details). Color scale represents steps of 0.1 microV.

Figure 3 shows the response-locked bandpass-filtered grand-averages for correct responses at the midline-electrode sites Fz, Cz and Pz, while figure 4 depicts the equivalent feedback-locked grand-averages. Grand averages as well as isovoltage maps of figure 3 indicate the presence of a small ERN for the LL condition only, i.e. the condition in which participants were most likely to have self-detected their excessive RT. The corresponding statistical analyses (repeated measures ANOVA with the factors RT-bin (levels: EA, EL and LL) and electrode site (levels: Fz, Cz, Pz)) revealed significant RT-bin main effects (response locked: F(2,32) = 8.6, p = 0.001, feedback locked: F(2,32) = 6.97, p = 0.006) and a significant RT-bin by electrode site interaction (response locked: F(4,64) = 11.91, p < 0.001, feedback locked: F(4,64) = 5.07, p = 0.005). Planed comparisons revealed significant differences between the LL and the EA and EL condition respectively (see table 1), while the comparison of the EA and EL conditions was not significant. While an obvious violation of the reaction time criterion elicited an ERN in the response locked data, the most pronounced feedback related negativity was observed in the EL condition (see figure 4), in which subjects obviously failed to detect a reaction time error by themselves and therefore had to fall back on feedback information. Planned comparisons revealed at Fz significant differences between EL and EA and LL conditions respectively, but not for the comparison of EA and LL, which had virtually identical waveforms within the pertinent time range (figure 4).

**Table 1:** T-values of planned comparisons

	Fz		Cz		Pz	
	response	feedback	response	feedback	response	feedback
EA vs. EL	0.01	6.47*	0.01	3.74	1.64	4.87 *
EA vs. LL	7.44*	0.01	7.44*	0.59	19.98**	3.53
EL vs. LL	5.17*	14.07**	5.17*	5.97*	7.80*	0.15

T-values of planned comparisons for ERN (response-locked) and FRN (feedback-locked) components; df = 16, * 0.05 < p < 0.01; ** p < 0.01.

**Figure 5:**
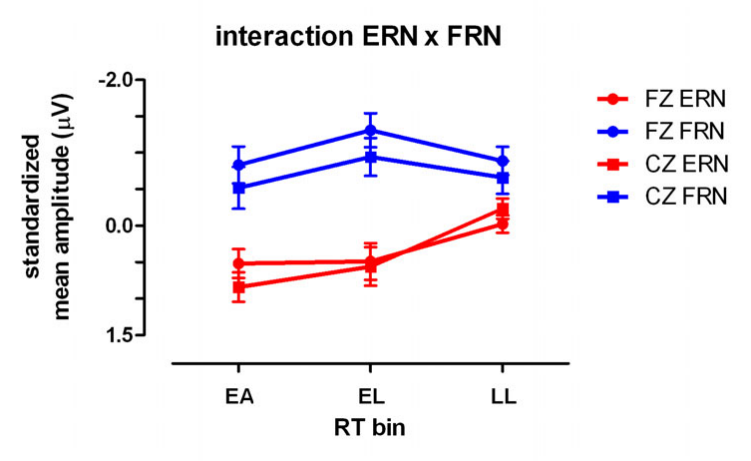
**Mean amplitudes of response and feedback locked ERPs**. Interaction plot for ERN and FRN amplitudes at electrode sites Fz and Cz. Error bars representing SEMs. Mean amplitudes based on McCarty-Wood standardized values.

Thus, it appears that a feedback related negativity occurred only in "early-late" cases that did not permit the self-detection of a time-out error, indicated by the absence of the ERN. To further address this point, an ERN/FRN × RT-bin × electrode site ANOVA was performed using the vector normalized data set. Importantly, a significant ERN/FRN × RT-bin interaction (F(2,32) = 7.58, p = 0.002) was obtained. This interaction is illustrated in figure 5 and supports an inverse ERN/FRN-relationship.

## Discussion

The data presented here indicate a functional interrelation of action monitoring processes which are assumed to underlie error and feedback related negativities: In cases in which self-generated information was sufficient to recognize a time-out error for sure an ERN was released, but no FRN was observed. This was true for the LL condition. By contrast, in the EL condition no ERN was seen but solely an FRN was observed. This suggests that action monitoring processes had to rely on externally provided information in this case. This pattern of results indicates that whenever feedback information is redundant, as in the case of the late late responses, it does not trigger a FRN.

This is in contrast to most studies investigating the nature of the FRN: typically, subjects performed different types of gambling tasks where the response indicates a choice between alternatives only. In this kind of paradigms the feedbacks' outcome, which provided crucial information about winning or losing, is not under control of the subjects' behavior [e.g. [15,18,20,21,24,33]]. Some studies also reported the appearance of a FRN without any related action [34]. The general reasoning of these studies was that the FRN reflects whether the outcome of an action was worse than expected. Importantly, the feedback stimulus was the only source of information. It is obvious that in these studies the robust release of an FRN only takes place, because subjects were unaware or unsure regarding the adequacy of their response. In one other study [25], however, an associative learning task was employed that demanded learning for a set of pictures whether a given picture required a left or a right button press. As over the course of an experimental run participants established stimulus-response mappings, the ERN to performance errors increased in amplitude, while at the same time the FRN to negative feedback decreased in amplitude. Importantly, this study already hinted at the fact, that FRN amplitude variations are inversely related to the predictability of an action's outcome. In the present study the expectation of an action's outcome was manipulated by the ability to detect an error. Whenever the error detection, indicated by the appearance of an ERN, was enabled based on self-generated information and according to this detected before the feedback presentation, the expectation corresponded with the real action's outcome, in the present study the feedback. No additional performance monitoring process was necessary and therefore, no FRN was released. If subjects were unsure about their performance or failed to detect an error, this condition was very similar to the experimental situation in the studies mentioned before. In such a case feedback stimuli indicated reliably an action's outcome worse than expected and a FRN was released. It is noteworthy that the only difference between the feedback in the EL and LL condition was the subjects' ability to detect a violation of the response deadline criterion before. Physically the feedback stimuli were identical.

A necessary prerequisite for showing this relationship was to establish a condition where a relevant criterion was violated without being noticed by the subject. Based on the known limitations of human beings to estimate their own reaction time precisely [12,35] this was achieved in the EL condition in which subjects made the correct button press but exceeded the response deadline criterion slightly. The absence of an ERN can be seen as a clear indication that action monitoring processes failed to detect this kind of errors. In contrast, if subjects detected a response speed error, like in the LL condition, a negative deflection is seen with the typical latency and topography of an ERN despite the correct button selection. The most pronounced ERN was related to incorrect, but in time button press responses. This pattern of results is in line with data reported by Luu and colleagues [11] and replicates their finding that action monitoring processes are able to consider more than one dimension concurrently. Luu et al. argued that ERN amplitude varies according to the degree of self-monitoring processes: the later the response, the stronger the demands for an attentional self-monitoring system and the larger the ERN amplitude. This argumentation is in line with a "certainty" account: the increase of the ERN is related to the subjective certainty of having made an error and is able to explain the pronounced ERN amplitude difference between the LL RT-bin and the incorrect button press shown in the present and the Luu et al. ([11] LATE condition) study: the incorrect button selection is obviously the most distinct type of error, while exceeding the response deadline is less obvious and therefore harder to detect by the subject [9,36].

Several studies [4] have shown that the ERN and concomitant error-related activations of the medial frontal cortex in fMRI can occur without subjective awareness of the error. With regard to the current set of data we therefore cannot assume that subjects are consciously aware of errors in the LL condition. This would need to be tested by a modified experiment. What we have shown is that "late late" time-out errors are internally detected by the action monitoring system resulting in a reduced impact of external feedback information in this kind of error.

## Conclusion

We have shown that the FRN is released only in cases where the feedback stimulus contains non-redundant information. This implies the release of a FRN depends also on results of those action monitoring processes, which are based on self-generated information and are executed before the appearance of feedback information.

## Abbreviations

ERN: Error related negativity; FRN: Feedback related negativity; ERP: Event-related potential; EA: Time bin early (in time); EL: Time bin early late; LL: Time bin late late; SD: Standard deviation; RT: Reaction time; ANOVA: Analysis of variance; fMRI: Functional magnetic resonance imaging.

## Authors' contributions

MH and THM conceived the experimental design. MH performed the study, made parts of the statistical analysis and wrote the first draft of the manuscript. JR performed parts of the statistical analysis. THM was involved in the EEG analysis and wrote parts of the final draft of this manuscript. All authors read and approved the final manuscript.
